# Bioactive Metabolite Production in the Genus *Pyrenophora* (Pleosporaceae, Pleosporales)

**DOI:** 10.3390/toxins14090588

**Published:** 2022-08-27

**Authors:** Marco Masi, Jesús García Zorrilla, Susan Meyer

**Affiliations:** 1 Department of Chemical Sciences, University of Naples “Federico II”, Complesso Universitario Monte S. Angelo, Via Cintia 4, 80126 Napoli, Italy; 2 Allelopathy Group, Department of Organic Chemistry, Facultad de Ciencias, Institute of Biomolecules (INBIO), University of Cadiz, C/Avenida República Saharaui, s/n, 11510 Puerto Real, Spain; 3 Shrub Sciences Laboratory, U.S. Forest Service Rocky Mountain Research Station, 735 North 500 East, Provo, UT 84606, USA

**Keywords:** *Pyrenophora*, toxins, biological activity, phytotoxicity, pathogenicity, biomolecules

## Abstract

The genus *Pyrenophora* includes two important cereal crop foliar pathogens and a large number of less well-known species, many of which are also grass pathogens. Only a few of these have been examined in terms of secondary metabolite production, yet even these few species have yielded a remarkable array of bioactive metabolites that include compounds produced through each of the major biosynthetic pathways. There is little overlap among species in the compounds identified. *Pyrenophora tritici-repentis* produces protein toxin effectors that mediate host-specific responses as well as spirocyclic lactams and at least one anthraquinone. *Pyrenophora teres* produces marasmine amino acid and isoquinoline derivatives involved in pathogenesis on barley as well as nonenolides with antifungal activity, while *P. semeniperda* produces cytochalasans and sesquiterpenoids implicated in pathogenesis on seeds as well as spirocyclic lactams with phytotoxic and antibacterial activity. Less well-known species have produced some unusual macrocyclic compounds in addition to a diverse array of anthraquinones. For the three best-studied species, in silico genome mining has predicted the existence of biosynthetic pathways for a much larger array of potentially toxic secondary metabolites than has yet been produced in culture. Most compounds identified to date have potentially useful biological activity.

## 1. Introduction

Species of the fungal ascomycete genus *Pyrenophora* are known to produce a spectacular array of secondary metabolites, but, to date, there has been no published effort to integrate the large volume of information available on this topic. In this review of bioactive metabolites produced by members of the genus, the goal is to present this information in a format that will be useful for agronomists studying plant disease and researchers in chemical ecology, as well as natural products chemists and applied scientists seeking novel compounds for diverse uses. 

*Pyrenophora* is a genus of approximately 190 currently recognized species in the Dothidiomycete family Pleosporaceae [1]. Both the family Pleosporaceae and the genus *Pyrenophora* are well supported as monophyletic groups based on molecular phylogenetic analysis, and the *Drechslera* anamorphs traditionally associated with *Pyrenophora* species are also supported as genetically similar to their teleomorphs and conspecific with them [2,3,4]. Most *Pyrenophora* species are foliar pathogens of grasses, but some are also known as endophytes, as foliar pathogens of dicots, and in at least one case, as a seed pathogen.

For many species, little information beyond a species description is available, but two economically important cereal crop foliar pathogens, *P. tritici-repentis* and *P. teres*, have been well-studied [5,6,7]. A third well-studied species is *P. semeniperda*, a seed pathogen under consideration as a possible biocontrol for weedy annual bromes [8,9]. This review covers the literature from 1934 through 2022. The toxic metabolites discovered in each of the three well-studied species are presented, along with a few reports from additional species, and their isolation, structure determination, and biological activities are discussed.

## 2. Biology, Pathogenicity, and Toxin Production of *Pyrenophora* spp.

### 2.1. Pyrenophora teres

#### 2.1.1. Biology and Pathogenicity of *Pyrenophora teres*

*Pyrenophora teres* is the causal agent of net blotch on barley, which is an economically important foliar disease that can cause up to 40% yield reduction and also lowers grain quality [7]. It reproduces sexually on standing barley at the end of the growing season and overwinters on crop residues. There are multiple cycles of asexual reproduction via conidia during the growing season. This pathogen can also infect developing seeds and be moved as seed-borne inoculum. It has long been known that there are two forms that are morphologically identical but that cause quite different disease symptoms on barley leaves. The net form *P. teres* f. *teres* (Ptt) causes longitudinal dark brown necrotic lesions that can later become chlorotic, while the spot form *P. teres* f. *maculata* (Ptm) causes circular or elliptical spots that are dark brown and are associated with chlorosis on the surrounding leaf tissues. The two forms are genetically distinct and may represent different species [10]. They can be induced to hybridize under laboratory conditions, but hybrids under natural conditions are extremely rare. The two forms also differ in the growth rate, symptom development, and toxin production in culture. 

*P. teres* can infect a wide range of cereal and native grass hosts, but rarely, if ever, causes significant disease on these hosts [11]. This makes wild grass species an unlikely source of inoculum. Disease levels on different barley cultivars appear to be mediated by gene-for-gene interactions, but toxins specifically produced by these virulence genes have not yet been identified [6,12]. Many of the toxins produced by this fungus in culture can induce some level of disease symptoms on barley leaves, but these effects are not strain- or host genotype-specific. 

Genome mining has identified a large number of predicted biosynthetic gene clusters that could mediate the production of novel toxins in *P. teres* [13]. The total number is greater for Ptt (36 to 82 depending on strain) than for Ptm (45–47, two strains). The majority of these (15–53) are NRPS (non-ribosomal peptide synthase) loci, with 12–15 PKS (polyketide synthase) loci, 2–9 PKS-NRPS hybrid loci, and 4–6 terpene biosynthase loci. There is a high probability that further research can unravel the identity of toxic effectors and their specificity in the gene-for-gene interactions that have been documented genetically and phenotypically in this pathosystem, whether the effectors are secondary metabolites or proteins [6].

#### 2.1.2. Phytotoxins Produced by *Pyrenophora teres*

**Figure 1:**
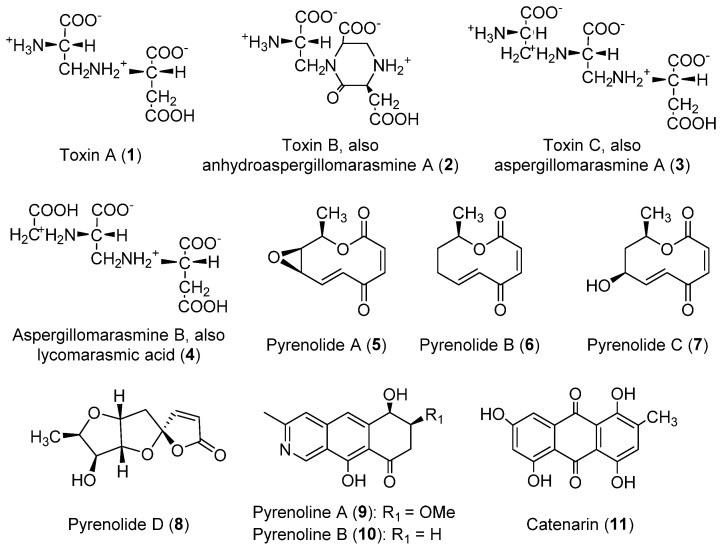
Structures of the toxins produced by *P. teres*.

Chemically diverse toxins have been isolated from cultures of *P. teres*. These compounds belong to different classes according to their structures, including amino acid derivatives of the marismine class, nonenolides, spirocyclic lactones, isoquinolines, and an anthraquinone (Figure 1). As reviewed in this section, many of them are phytotoxic, whereas other types of biological activities of agronomic or pharmacological interest have been described for some of them. 

Amino acid derivatives (**1**–**4**, Figure 1) in a family that belongs to the marasmine class are among the phytotoxins produced by *P. teres*. All of these were obtained from cultures grown in Fries’ liquid medium. Toxins A and B (**1** and **2**), isolated for the first time from culture filtrates of *P. teres* collected from barley leaves, were the first to be discovered from this family [14]. Their structures were described in a later study, with toxin A (**1**) being characterized as *N*-(2-amino-2-carboxyethyl)aspartic acid, and toxin B (**2**) as 1-(2-amino-2-carboxyethyl)-6-carboxy-3-carboxymethyl-2-piperazinone [15]. Toxin B (**2**) is also known as anhydroaspergillomarasmine A [16]. Compounds **1** and **2** showed phytotoxic effects on barley, provoking chlorosis and collapse of tissues [14]. It was suggested that toxins A and B (**1** and **2**) play a key role in the disease syndrome of net-spot blotch of barley, also contributing to the virulence of individual isolates of *P. teres* [14]. The same study that provided the characterization of compounds **1** and **2** [15] also reported the first isolation of toxin C (**3**) from *P. teres*. The structure of this compound, also known as aspergillomarasmine A, corresponded with that of *N*-[2-(2-amino-2-carboxyethylamino)-2-carboxyethyl]aspartic acid, an already-known fungal compound previously described from *Fusarium oxysporum*, *Colletotrichum gloeosporioides*, *Aspergillus flavus-oryzae,* and *Paecilomyces* species [16,17,18]. Toxin A (**1**) was suggested as a precursor of toxin C (**3**) in cultures of *P. teres*, whereas toxin C (**3**) generates toxin B (**2**) by a non-enzymatic mechanism [16]. Different strategies for the synthesis of compound **3** are available in the literature [19,20,21,22,23]. As for toxins A and B (**1** and **2**), toxin C (**3**) has phytotoxic activity on barley, and it has been suggested that compound **3** plays a major role in the pathological changes associated with the barley net-spot blotch disease [16]. Compound **3** also possesses pharmacological interest due to its activity against some factors that generate resistance in Gram-negative pathogens. In fact, it is a potent inactivator of metallo-β-lactamases and has proven to reverse carbapenem resistance in vivo [24]. The inhibition of metallo-β-lactamases by compound **3** would occur via the selective sequestering of Zn^2+^ [25]. Of interest for the development of drugs, it could be worth highlighting that the structure of compound **3** proved to be tolerant of changes in the stereochemistry at positions 3, 6, and 9 regarding the activity against the metallo-β-lactamase NDM-1 [20]. 

It is interesting to note the study by Weiergang et al. (2002) [26] on the phytotoxicity of toxins A–C (**1**–**3**) on barley leaves, which showed the different activity profiles of these compounds. It was found that toxin A (**1**) generates chlorotic symptoms and little necrosis, whereas toxin C (**3**) provokes distinct necrotic symptoms and chlorosis, and toxin B (**2**) is weakly toxic. It was concluded that the interaction of barley with toxins A and C (**1** and **3**) is correlated with that observed with *P. teres* (both *f. teres* and *f. maculata*). This suggested that these toxins may be used to select resistant barley lines in the early stages of breeding programs [26].

Aspergillomarasmine B (**4**), also known as lycomarasmic acid, is a toxin identified as a product of *P. teres* in 2008 [27]. This compound, similar to the closely related compound **3**, had been previously found in the fungal species *A. flavus-oryzae*, *C. gloeosporioides,* and *Paecilomyces* [17,18,28]. Its isolation from *C. gloeosporioides*, the pathogen of olive crops (*Olea europaea* L.), represented its first report as a toxin produced by a plant pathogen [28]. Compound **4** showed remarkable phytotoxic activity, whose mechanism may be based on a chelation process that forms toxic iron chelates [27].

The family of pyrenolides (**5**–**8**, Figure 1) constitutes a group of bioactive toxins also produced by *P. teres* that are compounds with antifungal activity [29] isolated from cultures grown in malt-dextrose medium. Structurally, pyrenolides **5**–**7** are nonenolides formed by a 10-membered lactone ring with different substituents. Pyrenolide A (**5**) was first isolated from *P. teres* [30]. It was later found in *Ascochyta hyalospora* [31], and some hydroxylated derivatives were isolated from a marine-derived *Curvularia* species [32]. Pyrenolides B and C (**6** and **7**) were isolated in a later study [29]. The synthesis of pyrenolide B (**6**) was reported in different studies [33,34,35], though not as an enantiomerically pure product. It is worth highlighting that Suzuki et al. (1987) [34] proved that synthetic (±)-pyrenolide B shows significant antimicrobial activity (against *Aspergillus niger* and *Cochliobolus miyabeanus*) and phytotoxicity. The synthesis of (±)-pyrenolide C by Wasserman and Prowse (1992) [36] was the first reported for this compound, also allowing the establishment of its stereochemistry. 

The structure of pyrenolide D (**8**) differs from those of the previously described pyrenolides A–C (**5**–**7**), showing a tricyclic spiro-γ-lactone scaffold of five-membered rings instead of the nonenolide scaffold. Pyrenolide D (**8**) was isolated for the first time from *P. teres* [37]. The same study reported that this compound possesses cytotoxic activity (against HL-60 cells), whereas antifungal activity was not found, unlike pyrenolides A–C. The synthesis of pyrenolide D (**8**) was the focus of later studies, as a result of which this toxin was obtained as an enantiomerically pure product [38,39,40,41,42].

Two isoquinolines, named pyrenolines A and B (**9** and **10**, Figure 1), were also reported as phytotoxins isolated from *P. teres* [43]. Both compounds showed phytotoxic activity on different plant species, including barley. Pyrenoline A (**9**) required lower concentrations to generate the phytotoxic effects evaluated. Pyrenoline A (**9**) did not show host specificity regarding monocot and dicot species. The kinetics of production of pyrenolines A and B (**9** and **10**) by *P. teres* were studied in later research. It was found that their concentration in the culture medium varies in time following a repetitive cycle of production and degradation, with pyrenoline B always being produced in higher quantities than pyrenoline A [44].

Catenarin (**11**) is a red anthraquinone pigment isolated from *Drechslera teres* [45] and other fungal species including *Helminthosporium gramineum*, *Pyrenophora tritici-repentis,* and *Conoideocrella krungchingensis* [46,47,48]. The culture medium employed for its isolation from *P. teres* is potato dextrose agar (PDA), unlike compounds **1**–**10**, for which a liquid medium (Fries, Malt-Dextrose or M1D) was used. Its synthesis was reported in the middle of the last century [48,49]. With regard to its biological activities, catenarin (**11**) induces necrosis on wheat in a non-specific manner [50] and inhibits, to some extent, the growth of the mycelium of *D. teres*, but not the germination of conidia [45]. Compound **11** also possesses a remarkable antibacterial profile. It significantly inhibits *B. subtilis* (at low concentrations of <0.1 µM) [45], as well as other Gram-positive bacteria and fungal species [46,50]. It is also cytotoxic against NCI-H187 cancer cells (IC_50_ = 8.21 µg/mL), and inactive against the non-cancerous line tested in the same assay [47]. Moreover, antidiabetic activity was described for catenarin (**11**), though few studies have been performed in this regard [51].

### 2.2. Pyrenophora tritici-repentis

#### 2.2.1. Biology and Pathogenicity of *P. tritici-repentis*

*Pyrenophora tritici-repentis* is the causal agent of the foliar disease tan spot of wheat [5]. It also occurs on related cereal crops and some native grasses but is not known to cause serious disease on these hosts. It is a necrotrophic pathogen that can survive saprophytically and increase its inoculum through sexual reproduction on crop residues over winter. Recent research on this disease has focused on the role of host-specific toxins (HSTs) in the pathogenesis of different cultivars of wheat. HST genes interact with host sensitivity genes in a manner that is essentially the inverse of the interaction of avirulence genes in biotrophic fungi with host resistance genes. In biotrophs, the host resistance gene product can recognize the pathogen avirulence gene product and initiate defense measures, including programmed cell death, that prevent further tissue colonization by the pathogen. However, for necrotrophic pathogens, programmed cell death is the opening that enables successful infection; thus, recognition by the host actually increases pathogen virulence. There are currently three HSTs known to be produced by this pathogen on wheat, and the combination of these in any pathogen strain and their complementary sensitivity genes in the host determines which wheat cultivars are susceptible to a given strain.

Tan spot disease has long been endemic in wheat but was considered a minor pathogen until quite recently. It has emerged as an economically important disease of wheat worldwide only in the last 60–80 years [52]. The advent of no-till agriculture is one probable contributor to its recent ascendance as a major disease of wheat. However, the major factor, as discussed below, that has increased its virulence on wheat involved a recent horizontal gene transfer from a related wheat pathogen, *Stagonospora nodorum* [53]. Both organisms produce PtrToxA, a host-specific toxin (HST) that causes severe disease in wheat cultivars that possess the corresponding sensitivity gene *Tsn1.* PtrToxA-producing strains have now become the prevalent strains in wheat-producing regions across most of the world (e.g., [54]). Even more recently, there appears to have been a second horizontal gene transfer of the PtrToxA gene from *P. tritici-repentis* to *P. teres*, which has enabled this barley pathogen to effectively expand its host range to include wheat [55]. Horizontal gene transfer is difficult to demonstrate conclusively, but the evidence *for PtrToxA* horizontal gene transfer into *P. tritici-repentis* is quite strong. 

*P. tritici-repentis* also produce toxins that are not host-specific, but there has been little research on the role of these toxins in disease development in wheat. A genome-mining exercise for this pathogen revealed the presence of >30 putative genes or gene clusters that are likely responsible for the biosynthesis of some of these other toxins [5]. These included both NRPS (non-ribosomal peptide synthase) and PKS (polyketide synthase) loci as well as two NRPS-PKS hybrid loci. More recently, a more comprehensive genome mining exercise identified a similar number of these biosynthesis gene clusters in *P. tritici-repentis*, as well as a number of terpene synthesis clusters [13]. The NRPS-PKS biosynthesis gene cluster responsible for triticone (spirostaphylotrichin) biosynthesis has been specifically identified [56].

#### 2.2.2. Phytotoxins Produced by *Pyrenophora tritici-repentis*

**Figure 2:**
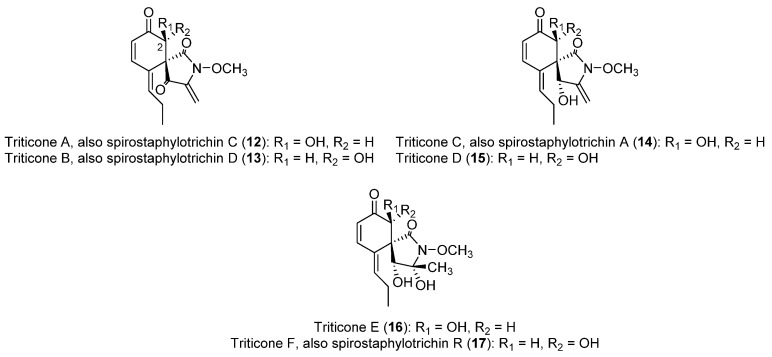
Structures of triticones A-F, toxins produced by *P. tritici-repentis*.

For the species *P. tritici-repentis*, a collection of toxins, mostly with protein- and spirocyclic lactam-like structures (Figure 2), has been isolated and studied.

The most studied toxins produced by *P. tritici-repentis* are the proteins known as Ptr ToxA and Ptr ToxB, obtained from cultures grown in Fries’ medium. They are host-selective toxins reported as necrosis-inducing in the case of Ptr ToxA [57], and chlorosis-inducing in the case of Ptr ToxB [58]. Ptr ToxA causes quicker symptoms than Ptr ToxB, though the defense responses observed have multiple similarities [59]. It was also found that Ptr ToxB has a greater distribution than the common host-selective toxins [60]. Pandelova et al. [59] provided an excellent overview of the biochemical mechanisms and effects of both toxins. 

Ptr ToxC was also reported as a chlorosis-inducing and low-molecular-weight compound, grown in a PDA medium [61,62]. This compound has a difficult isolation process and is not stable; its genetics are still under study. It has recently been suggested that it is not a protein [63]. 

As mentioned earlier, a family of spirocyclic lactams (**12**–**17**), named triticones or spirostaphylotrichins (Figure 2), has been described as toxins produced by *P. tritici-repentis* [64]. All of these were obtained from cultures grown in an M1D-modified liquid medium. Triticones A and B (**12** and **13**, Figure 2), epimeric compounds at C-2, were the first to be reported, isolated in 1988 as new chemotypes for which no closely related molecules had been described [65]. This study highlighted the instability of the active fractions to high temperatures and silica gel, making possible the isolation of the compounds by crystallization after the slow evaporation of the solvent. The ratio of production is approximately 1:1 [64]. Another relevant finding is that triticone A (**12**) undergoes racemization to form triticone B (**13**), and vice versa, which means that studies on the bioactivities of these compounds are commonly carried out on mixtures of both compounds. 

Triticones A and B (**12** and **13**) showed remarked phytotoxicity in leaf assays [64], and also showed phytotoxic activity at 4.0 µM in a wheat protoplast assay [65]. The mixture of triticones A and B induces chlorosis and necrosis on a wide range of monocot and dicot plants [56,66] and also inhibits CO_2_ fixation by 50% in wheat at 32 ± 13 µM [66]. Antibacterial activity against the Gram-positive species *Bacillus subtilis* and *Rhodococcus erythropolis* was reported, whereas no activity was observed against different Gram-negative bacteria or fungal species [56]. Triticone B (**13**) showed attributes of pharmacological interest, as it enhances plasmin activity of bovine aortic endothelial cells, causing direct and reversible inhibition of plasminogen activator inhibitor-1 [67].

As for triticones A and B (**12** and **13**), triticones C and D (**14** and **15**, Figure 2) were also described as epimers at C-2, and this is also the case for triticones E and F (**16** and **17**, Figure 2) [64,66]. Interestingly, triticones C and D (**14** and **15**) do not undergo the quick interconversion previously described for triticones A and B [64]. Unlike triticones A and B (**12** and **13**), which possess a marked phytotoxicity, triticones C and D (**14** and **15**) are weakly phytotoxic in leaf protoplast assays, whereas triticones E and F (**16** and **17**) are essentially inactive [64]. On the other hand, it is worth highlighting that these two latter compounds, in a mixture of 2:1, possess antibacterial activity against *Escherichia coli* (minimum inhibitory concentration = 62.5 μg/mL) [68].

Catenarin (**11**, Figure 1), a toxin produced by *P. teres* with phytotoxic and diverse pharmacological activities as previously described in Section 2.1.2, is also produced by *P. tritici-repentis* [46]. A study on *P. tritici-repentis* reported that the highest catenarin concentrations can be obtained in the Fries medium supplemented with starch. It was also shown that in specific conditions of incubation, a rapid accumulation of catenarin can occur during the first week, followed by a large decline after 14 days. This indicates that it may be bio-transformed to other anthraquinones or incorporated into melanin [50,69]. 

### 2.3. Pyrenophora semeniperda

#### 2.3.1. Biology and Pathogenicity of *Pyrenophora semeniperda*

*P. semeniperda* (alternate spelling *P. seminiperda*) is a generalist seed pathogen that attacks seeds in field seed banks [70]. It is known almost entirely from its anamorph *Drechslera campanulata*, as the sexual state is very rarely observed in nature and nearly impossible to obtain in culture [71]. The fungus forms macroscopic fingerlike stromata that protrude from killed seeds, earning it the moniker ‘black fingers of death’. Early studies on this pathogen in Australia addressed its potential as a biocontrol for annual grass weeds [72]. This has also been the motivating force behind extensive studies on the biology of this species in semiarid North America [8,9]. The Australian studies were initially based on the inoculation of non-dormant seeds, a treatment that resulted in very low seed mortality. These workers surmised that floral infection during seed development must account for the high natural mortality in soil seed banks of these weeds, and they demonstrated experimentally that this was at least possible [73]. Working with the host *Bromus tectorum* in the US, it was later discovered that the inoculation of mature seeds could cause very high mortality if seeds were inoculated when dormant [74]. Non-dormant seeds could escape mortality as in the Australian studies. Non-dormant *B. tectorum* seeds could also be killed in field seed banks under conditions of water stress that retarded seed germination but permitted pathogen activity [75]. 

In studies with multiple strains, it was discovered that slower-growing strains were better able to kill non-dormant *B. tectorum* seeds than fast-growing strains [76]. This was interpreted as a trade-off between the growth rate and production of cytochalasin B, a toxin produced in abundance by this pathogen [77]. This hypothesis was later confirmed experimentally [78].

Molecular genetic studies showed that *P. semeniperda* exhibits high levels of genetic diversity and regional genetic differentiation, even at the ITS locus, which is most often monomorphic at the species level [79]. It was later demonstrated that strains with different ITS haplotypes are strongly genetically differentiated and likely represent cryptic species [80].

Studies on the host range of this seed pathogen determined that it has a very wide host range, but that some hosts were more susceptible than others [81]. Reciprocal inoculation experiments with strains from different annual grass hosts demonstrated a complete lack of host specialization [82]. Strains varied in virulence and host species varied in resistance, but there was no pattern of increased virulence in the host of origin. 

A provisional genome mining exercise (C. Coleman, Brigham Young University, unpublished data) using an annotated genome assembly [83] yielded 12 predicted PKS loci, 8 predicted NRPS loci, and 2 PKS-NRPS hybrid loci. The two hybrid loci were later determined to be responsible for the biosynthesis of cytochalasins and spirostaphylotrichins, both of which are known to be produced by this fungus in culture.

#### 2.3.2. Phytotoxins Produced by *Pyrenophora semeniperda*

**Figure 3:**
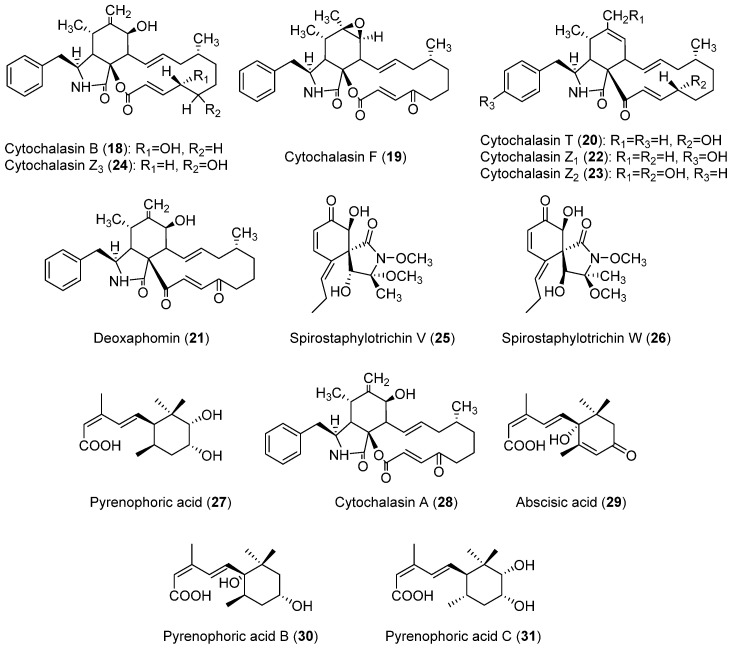
Structures of the toxins produced by *Pyrenophora semeniperda*.

*P. semeniperda* is a species for which a higher diversity of compounds (Figure 3) has been found, in comparison to *P. teres* and *P. tritici-repentis*. They include cytochalasan, spirocyclic lactam, and sesquiterpenoid acid structures. Interestingly, some of the compounds produced by *P. semeniperda* have been also discovered in *P. tritici-repentis*, i.e., triticones A–C and E–F (**12**–**14**, **16** and **17**, Figure 2), previously described in Section 2.2.2. These will be referred as spirostaphylotrichins in this section when possible, as they were designated as spirostaphylotrichins in subsequent publications on *P. semeniperda*. Cytochalasins B, F, T, and deoxaphomin (**18**–**21**, Figure 3), as well as the previously undescribed cytochalasins Z1, Z2, and Z3 (**22**–**24**, Figure 3), were isolated in 2002 by Evidente et al. as the first phytotoxins produced on solid wheat culture by an Australian strain of *P. semeniperda* [84]. They belong to the cytochalasan group of fungal polyketide-amino acid hybrid metabolites with several biological activities [85,86]. Cytochalasins Z1, Z2, and Z3 (**22**–**24**) were characterized as 24-oxa[14]cytochalasans by NMR and MS techniques. Compounds **18**–**24** were assayed on wheat and tomato seedlings, and the most active compounds proved to be cytochalasin B (**18**), F (**19**), Z3 (**24**), and deoxaphomin (**21**). These showed a remarkable ability to inhibit root elongation. In leaf-puncture assay, only deoxaphomin (**21**) showed the ability to produce small necrotic lesions, whereas no effects were observed in the immersion assay from any of the tested cytochalasins [84].

Preliminary in vitro experiments showed that the fungus was able to produce other low-molecular-weight lipophilic phytotoxins in liquid culture, but they were not characterized [84,87]. These metabolites were identified as spirocyclic γ-lactams by Masi et al. [88] working with the PDB liquid cultures of a *P. semeniperda* strain collected in the USA. In particular, this strain produced the known spirostaphylotrichins A, C, D, R (**12**–**14** and **17**, Figure 2) and V (**25**, Figure 3), as well as triticone E (**16**, Figure 2), and a previously undescribed related compound, which was named spirostaphylotrichin W (**26**, Figure 3). The structure of this latter compound, as well as its relative stereochemistry, was characterized by spectroscopic and chemical methods. All the isolated compounds were tested in a *B. tectorum* coleoptile bioassay at a concentration of 10^−3^ M. Spirostaphylotrichin A (**12**) proved to be the most active compound, followed by spirostaphylotrichins C and D (**13** and **14**). Furthermore, in a leaf puncture bioassay carried out on host and non-host plants, only spirostaphylotrichins A, C, and D (**12**–**14**) exhibited phytotoxicity [88]. When the same strain was grown in solid culture on wheat culture, cytochalasin B (**18**) was identified as the main metabolite. Its production by other strains was also evaluated using a high-pressure liquid chromatography method (HPLC). This study revealed that the production of cytochalasin B (**18**) is strongly dependent on cultural conditions and that it is produced in large quantities in solid wheat seed culture (with production varying from 535 to 2256 mg kg^−1^). Furthermore, in a *B. tectorum* coleoptile bioassay, solid culture extracts of the strain studied showed higher toxicity than the cytochalasin B standard at the highest concentration tested. This suggested the possible presence of other phytotoxic metabolites in the organic extracts [77]. 

Thus, the organic extract of *P. semeniperda* strain WRR10-16, one of the most active strains in the *B. tectorum* coleoptile bioassay [89], was purified using different steps of column chromatography, also yielding the other known cytochalasins F and Z3 (**19** and **24**) and deoxaphomin (**21**), as well as a previously undescribed sesquiterpenoid penta-2,4-dienoic acid that was named pyrenophoric acid (**27**, Figure 3) [89]. Its relative stereochemistry was assigned by NMR studies while its absolute configuration was determined by applying the advanced Mosher’s method [90]. Pyrenophoric acid (**27**) proved to be very phytotoxic in a cheatgrass coleoptile elongation test at 10^−3^ M and its negative effect on coleoptile elongation was additive with that of cytochalasin B when tested in a mixture at 10^−4^ M. This result demonstrated that the high toxicity shown by the organic extract was due to the combined action of multiple phytotoxic compounds [89]. 

When the same fungus was grown in cheatgrass seed culture, two other previously undescribed compounds were isolated together with cytochalasins A, B, F, and Z3 (**28**, **18**, **19** and **24**, respectively, Figure 3), deoxaphomin, pyrenophoric acid, and abscisic acid (**21, 27** and **29**, respectively, Figure 3). The two new compounds that were characterized by spectroscopic methods and, as they were related to pyrenophoric acid, were named pyrenophoric acids B and C (**30** and **31**, Figure 3). In a cheatgrass seedling bioassay at 10^−3^ M, pyrenophoric acid B (**30**) showed higher coleoptile toxicity than pyrenophoric acid, while pyrenophoric acid C (**31**) showed lower phytotoxicity [91]. 

Another study demonstrated that the production of cytochalasin B (**18**) could also be induced in liquid media only if they contained host seed constituents. This strongly suggests that the production of cytochalasin B is directly implicated in the pathogenesis of seeds [78]. Research on the mode of action of pyrenophoric acid B (**30**) using mutant lines of *Arabidopsis thaliana* demonstrated that this compound activates the abscisic acid (ABA) signaling pathway in order to inhibit seed germination. It was demonstrated that it uses the ABA biosynthesis pathway at the level of alcohol dehydrogenase ABA2 to achieve this inhibition. This result suggested that *P. semeniperda* may manipulate plant ABA biosynthesis in the seed as a strategy to reduce germination, increasing its ability to cause seed mortality and thereby increase its fitness through higher reproductive success [92].

### 2.4. Other Pyrenophora *spp.*

#### 2.4.1. Biology and Pathogenicity of other *Pyrenophora* Species

Many other *Pyrenophora* species are foliar grass pathogens with life histories similar to *P. teres* and *P. tritici-repentis*, and this is especially true of those that have been studied in terms of secondary product chemistry. As these pathogens are less economically important, their biology and pathogenicity have received much less attention. Four species have been investigated to varying degrees for toxin production: *P. avenae* (syn. *P. chaetomioides*, anamorph *D. avenae*), *P. lolii* (anamorph *D. siccans*), *P. catenaria* (anamorph *D. catenaria*), and *P. biseptata* (anamorph *D. biseptata*). *Pyrenophora avenae* is primarily a disease of cultivated oats [93,94] while *P. lolii* infects cultivated and wild species of *Lolium* (ryegrass; [95,96]). Little information is available on the biology of the other two species. There is a report on secondary product chemistry for *D. dematioidea* as an endophyte in a species of marine algae [97], but as this identification was based only on morphology in a group where even the generic boundaries are not clear [98,99], we have chosen not to include this paper in our survey of toxin production in *Pyrenophora*.

#### 2.4.2. Phytotoxins Produced by other *Pyrenophora* spp.

**Figure 4:**
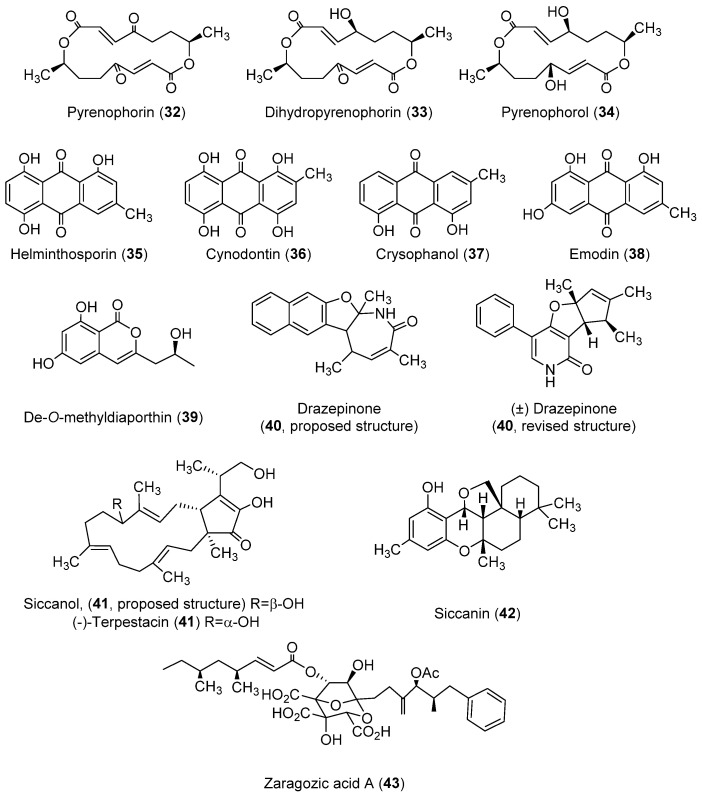
Structures of toxins produced by other *Pyrenophora* species.

As reviewed in Section 2.1, Section 2.2 and Section 2.3, diverse families of toxins with different structures have been isolated from *P. teres*, *P. tritici-repentis,* and *P. semeniperda*. Nevertheless, markedly different toxins have been isolated from other *Pyrenophora* species (Figure 4). These toxins are reviewed in this section.

*Pyrenophora avenae*, a pathogen of oats, produces toxins with macrocyclic and anthraquinone structures. The toxins with a macrocyclic structure produced are pyrenophorin (**32**, Figure 4) [100] and the structurally related compounds dihydropyrenophorin and pyrenophorol (**33** and **34**, Figure 4) [101]. Pyrenophorin (**32**) inhibited radicle growth in oat and non-host plants [102]. This toxin has antifungal properties, as it is significantly active against the biotrophic pathogen *Microbotryum violaceum* and the yeast *Saccharomyces cerevisiae* at 5 µM [103]. Moreover, pyrenophorin (**32**) showed strong cytotoxicity against several cancer cell lines (IC_50_ values ranging from 0.07 to 7.8 μM) [104]. The stereoselective total synthesis of pyrenophorin has been published [105]. Dihydropyrenophorin (**33**) showed phytotoxic activity [101], as well as antibacterial, antifungal, and antialgal activities [106]. These last antimicrobial activities were also found for pyrenophorol (**34**) [101,106]. Compound **34** showed phytotoxicity (leaf necrosis) on *Avena sterilis* and, at a lower level, on *Avena fatua* L. On the other hand, the seed germination and seedling growth of *A. sterilis* were not affected [107]. The stereoselective total synthesis of pyrenophorol has been published [108].

In regard to the toxins with an anthraquinones structure produced by *P. avenae*, these compounds are helminthosporin and cynodontin (**35** and **36**, Figure 4), two metabolites produced by diverse fungal species. As for the previously described anthraquinone catenarin (**11**), the growth medium was PDA [45], while Czapek-Dox was also employed as a medium for obtaining compound **35 [109]**. Helminthosporin (**35**) is a toxin that showed herbicidal activity against different weed and crop plants, though species such as soybean, tomato, or cotton were resistant when tested at 500 µg/mL [110]. Compound **35** also showed positive results in pharmacological assays. It inhibited the growth of hepatic bile duct (TFK-1) and liver (HuH7) cancer cell lines [111] and also showed significant inhibition of electric eel acetylcholinesterase (IC_50_ = 2.53 µM) and brain permeable properties [112]. In the case of cynodontin (**36**), relevant antifungal activity was found against *Sclerotinia minor*, *Sclerotinia sclerotiorum*, and *Botrytis cinerea* [113]. It is worth highlighting the study by Đorović et al. [114], which examined the antioxidative mechanisms of action of cynodontin.

Three relevant anthraquinones were also isolated from the species *Drechslera catenaria* (grown in Czapek-Dox medium), named chrysophanol and emodin (**37** and **38**, Figure 4), as well as the already-described catenarin (**11**, Figure 1, Section 2.1.2) [115]. Chrysophanol (**37**) possessed poor phytotoxic activity, as tested on *Arabidopsis thaliana* [116], although it showed antifungal properties, including against plant pathogenic fungi [117]. Indeed, curative and protective activity against barley powdery mildew was demonstrated [118]. On the other hand, chrysophanol (**37**) has remarkable pharmacological potential, as recently reviewed by Yusuf et al. (2019) [118] and Su et al. (2020) [119]. Particularly, this compound showed anti-inflammatory, antiviral, anti-cancer, neuroprotective, anti-cardiovascular disease, and anti-ulcer activities. Research on the pharmacological bioactivities of chrysophanol (**37**) continues to be a topical issue. As examples of recent discoveries, the findings on its role in protecting against acute kidney injury [120], autologous blood-induced intracerebral hemorrhage [121], and in vivo hippocampal damage and mitochondrial autophagy [122] could be highlighted. Regarding emodin (**38**), it has been traditionally used in Chinese medicine, with a wide spectrum of later-proven pharmacological activities, but also adverse effects when used long-term at high doses [123]. This compound is the direct precursor of catenarin (**11**, Figure 1) [124] and is also a phytotoxin. It was found to have inhibitory activity on sunflowers (*Helianthus annuus*) [125] and the weeds *Amaranthus hypochondriacus* and *Echinochloa crus-galli* [126].

Toxins with diverse types of structures have been found for the pathogen *Drechslera siccans* (**39**–**42**, Figure 4) through the use of the liquid growth medium M1D modified, or glucose-potato broth-agar in the case of compound **42**. De-*O*-methyldiaporthin (**39**) is phytotoxic to barnyard grass, corn, and soybean, though poor or null activity was found for host plants of *D. siccans* [127]. Drazepinone (**40**) was isolated as a new phytotoxic trisubstituted naphthofuroazepinone, though its structure was recently revised (see **40**, Figure 4) [128,129]. This compound causes necrosis in a wide range of plant species, with *Urtica dioica* L. being the most affected tested species [128]. It also showed protein tyrosine phosphatase inhibitory activity [129] but low zootoxicity [128]. Siccanol (**41**), a bicyclic sesquiterpene that showed phytotoxicity on the root growth of Italian ryegrass (*Lolium multiflorum,* a *D. siccans* host plant), was also isolated from *D. siccans* [130]. Its structure was revised and assigned as (-)-terpestacin based on the total synthesis of this compound, which was isolated from other fungal species [131,132]. Siccanin (**42**), another toxin isolated from *D. siccans*, was active against *Trichophyton* [133]. Inhibitory activity to succinate dehydrogenase was also found (IC_50_ = 0.9 µM) [134]. Its total synthesis was reported [135]. 

Finally, it is worth highlighting zaragozic acid A (**43**), also known as squalestatin S1, a toxin produced by *Drechslera biseptata*. Although few references have been published on its activity, squalene synthase inhibitor activity was described [136,137]. The synthesis of this compound was also accomplished [138].

## 3. Classification of the Toxins Produced by *Pyrenophora* spp. according to Their Structures

In order to provide a clear overview in relation to the structures, origin, and biological activities described for the compounds under review (**1**–**43**), Table 1 compiles this information through a classification of the compounds according to their chemical classes.

This classification highlights how phytotoxic activity, whether detected for host plants or other species, has been shown by the vast majority of classes of compounds produced. This result, obtained after numerous studies carried out over decades, emphasizes the interest that exists in continuing with the study of the genomic aspects and modes of action involved in the phytopathogenic *Pyrenophora* species. Likewise, finding phytotoxic compounds could provide new herbicides based on natural products. A priori, they could present the advantages of reducing environmental impact, requiring lower doses of the active compound, or applying alternative modes of action to conventional herbicides, thus avoiding resistance problems. However, a significant difficulty is that the isolation of the toxins from natural sources often has excessively low yields. For this reason, throughout this review, the most outstanding publications on the synthesis of some of these toxins have been highlighted.

This discussion can be extrapolated to the pharmacological field, given the activity shown by some of the toxins in tests for antimicrobial or cytotoxic effects. In this regard, available references on pharmacological activities are provided for anthraquinones, cytochalasans, and macrocyclic or spirocyclic compounds. The anthraquinone chrysophanol (**37**) represents one of the most studied. It was noted in a recent review that relevant aspects of its mechanism of action and pharmacokinetics are still unknown [118].

## 4. Conclusions

The research to date on toxin production in the genus *Pyrenophora* described here has likely only scratched the surface in terms of the potential of members of this genus to produce novel and interesting toxic compounds. First, very few species have been investigated, and there is remarkably little overlap among study species in the compounds produced. Of the several classes of compounds detected, only the spirocyclic lactams were common to both *P. tritici-repentis* and *P. semeniperda*, and the only other compound common to multiple species was the anthraquinone catenarin. The unusual compounds produced by economically unimportant *Pyrenophora* species were especially noteworthy. Another indication that many potential compounds have gone undetected is the large number of predicted biosynthesis genes from in silico analyses of the three well-studied species that have no known corresponding gene products. New molecular tools may make it possible to induce the production of some of these secondary metabolites in vitro so that they can be characterized and understood [144]. In the meantime, traditional approaches to the discovery of new secondary metabolites, in *Pyrenophora* and perhaps in general, are more likely to be successful if they are focused on understudied fungal pathogens from non-agronomic systems. 

## Figures and Tables

**Table 1:** Classification of the toxins (**1**–**43**) according to their chemical classes.

Class	Compound	*Pyrenophora* species	Activity	References
Amino acid derivatives	Toxin A [*N*-(2-amino-2-carboxyethyl) aspartic acid] (**1**, Figure 1)	*P. teres*	Phytotoxic to barley	[14,15,16,26,27,139]
	Toxin B [1-(2-amino-2-carboxyethyl)-6- carboxy-3-carboxymethyl-2-piperazinone]; anhydroaspergillomarasmine A (**2**, Figure 1)	*P. teres*	Phytotoxic to barley	[14,15,16,26,27,139]
	Toxin C [*N*-[2-(2-amino-2-carboxy ethyl-amino)-2-carboxyethyl] aspartic acid]; aspergillomarasmine A (**3**, Figure 1)	*P. teres*	Phytotoxic to barley; reverse of resistance to Gram-negative pathogens	[15,16,24,26,27,139]
	Aspergillomarasmine B; lycomarasmic acid (**4**, Figure 1)	*P. teres*	Phytotoxic to barley	[27]
Anthraquinones	Catenarin (**11**, Figure 1)	*P. catenaria* *P. teres* *P. tritici-repentis*	Phytotoxic to wheat; antibacterial; antifungal; cytotoxic; antidiabetic	[45,46,47,50,51,109,115]
	Chrysophanol (**37**, Figure 4)	*P. catenaria*	Antifungal; anti-inflammatory; antiviral; anti-cancer; neuroprotective; anti-cardiovascular disease; antiulcer	[115,117,118,119,120,121,122]
	Cynodontin (**36**, Figure 4)	*P. avenae*	Antifungal; antioxidant	[45,109,113,114]
	Emodin (**38**, Figure 4)	*P. catenaria*	Phytotoxic to sunflower, *Amaranthus hypochondriacus* and *Echinochloa crus-galli*; antibacterial; anticancer; hepatoprotective; anti-inflammatory; antioxidant; antimicrobial	[115,123,124,125,126]
	Helminthosporin (**35**, Figure 4)	*P. avenae* *P. catenaria*	Herbicidal; cytotoxic; inhibition of cholinesterase	[45,109,110,111,112]
Bicyclic sesquiterpene	Siccanol; (-)-terpestacin (**41**, Figure 4)	*D. siccans*	Phytotoxic to *Lolium multiflorum*	[130]
Cytochalasans	Cytochalasin A (**28**, Figure 3)	*P. semeniperda*	Phytotoxic to *Bromus tectorum, Cirsium arvense* and *Sonchus* *arvensis*; anticancer; antibacterial; antifungal; antiviral	[85,86,91,140,141]
	Cytochalasin B (**18**, Figure 3)	*P. semeniperda*	Phytotoxic to wheat, tomato, *B. tectorum*, *Lilium longiflorum, C. arvense* and *S.* *arvensis*; algicidal; anticancer; cytotoxic; antiparasital; enzyme inhibition	[77,78,84,85,86,89,91,140,141,142]
	Cytochalasin F (**19**, Figure 3)	*P. semeniperda*	Phytotoxic to wheat, tomato, *B. tectorum*, *C. arvense* and *S.* *arvensis*; algicidal; anticancer	[84,85,86,89,91,140,141,142]
	Cytochalasin T (**20**, Figure 3)	*P. semeniperda*	Phytotoxic to *C. arvense* and *S. arvensis*	[84,141]
	Cytochalasin Z1 (**22**, Figure 3)	*P. semeniperda*	-	[84]
	Cytochalasin Z2 (**23**, Figure 3)	*P. semeniperda*	Phytotoxic to *C. arvense* and *S.* *arvensis*	[84,141,142]
	Cytochalasin Z3 (**24**, Figure 3)	*P. semeniperda*	Phytotoxic to wheat, tomato, *C. arvense* and *S.* *arvensis*; anticancer	[84,89,91,140,141,142]
	Deoxaphomin (**21**, Figure 3)	*P. semeniperda*	Phytotoxic to *B. tectorum,* s *C. arvense* and *S.* *arvensis*; anticancer	[84,89,91,140,141,142]
Isocoumarin	De-*O*-methyldiaporthin (**39**, Figure 4)	*D. siccans*	Phytotoxic to corn, soybean, *Amaranthus spinosus*, *Digitaria ischaemum* and *E. crus-galli*	[127]
Isoquinoline derivatives	Pyrenoline A (**9**, Figure 1)	*P. teres*	Phytotoxic to barley, *Festuca* spp., *Agropyron repens* and *Cynodon dactylon*	[43]
	Pyrenoline B (**10**, Figure 1)	*P. teres*	Phytotoxic to barley, oat, *Hibiscus sabdariffa* and *Euphorbia heterophylla*	[43]
Macrocyclic compounds	Pyrenophorin (**32**, Figure 4)	*P. avenae*	Inhibition of radical growth in oat and non-host plants; antifungal; cytotoxic	[100,102,103,104]
	Dihydropyrenophorin (**33**, Figure 4)	*P. avenae*	Phytotoxic to barley, soybean, wheat, maize, oat, *Sorghum halepense* and different weeds; antibacterial; antifungal; antialgal	[101,106]
	Pyrenophorol (**34**, Figure 4)	*P. avenae*	Phytotoxic to oat and tomato; antibacterial; antifungal; antialgal	[106,107,143]
Naphthofuroazepinone	Drazepinone (**40**, Figure 4)	*D. siccans*	Phytotoxic to durum wheat and diverse weed species; protein tyrosine phosphatase inhibitor	[128,129]
Nonenolides	Pyrenolide A (**5**, Figure 1)	*P. teres*	Antifungal	[30]
	Pyrenolide B (**6**, Figure 1)	*P. teres*	Antifungal	[29]
	Pyrenolide C (**7**, Figure 1)	*P. teres*	Antifungal	[29]
Phenolic compound	Siccanin (**42**, Figure 4)	*D. siccans*	Antifungal; succinate dehydrogenase inhibition	[133,134]
Proteins	Ptr ToxA	*P. tritici-repentis*	Phytotoxic to wheat	[57]
	Ptr ToxB	*P. tritici-repentis*	Phytotoxic to wheat	[58]
Sesquiterpenoids	Abscisic acid (**29**, Figure 3)	*P. semeniperda*	Phytotoxic to *B. tectorum*	[91,92]
	Pyrenophoric acid (**27**, Figure 3)	*P. semeniperda*	Phytotoxic to *B. tectorum*	[89,91,92]
	Pyrenophoric acid B (**30**, Figure 3)	*P. semeniperda*	Phytotoxic to *Arabidopsis thaliana* and *B. tectorum*	[91,92]
	Pyrenophoric acid C (**31**, Figure 3)	*P. semeniperda*	Phytotoxic to *B. tectorum*	[91,92]
Spirocyclic lactams	Triticone A; spirostaphylotrichin C (**12**, Figure 2)	*P. semeniperda* *P. tritici-repentis*	Phytotoxic to wheat, tomato, oat, and different weed species	[64,65,66,88]
	Triticone B; spirostaphylotrichin D (**13**, Figure 2)	*P. semeniperda* *P. tritici-repentis*	Phytotoxic to wheat, tomato and different weed species	[64,65,88]
	Triticone C; spirostaphylotrichin A (**14**, Figure 2)	*P. semeniperda* *P. tritici-repentis*	Phytotoxic to *B. tectorum* coleoptiles, weakly to wheat, tomato and different weed species	[64,66,88]
	Triticone D (**15**, Figure 2)	*P. tritici-repentis*	Weakly phytotoxic to wheat and different weed species	[64,66]
	Triticone E (**16**, Figure 2)	*P. semeniperda* *P. tritici-repentis*	Antibacterial	[64,68,88]
	Triticone F; spirostaphylotrichin R (**17**, Figure 2)	*P. semeniperda* *P. tritici-repentis*	Antibacterial	[64,68,88]
	Spirostaphylotrichin V (**25**, Figure 3)	*P. semeniperda*	Weakly phytotoxic to *B. tectorum* coleoptiles	[88]
	Spirostaphylotrichin W (**26**, Figure 3)	*P. semeniperda*	Weakly phytotoxic to tomato and *B. tectorum* coleoptiles	[88]
Spirocyclic lactone	Pyrenolide D (**8**, Figure 1)	*P. teres*	Cytotoxic	[37]
Squalestatin	Zaragozic acid A; squalestatin S1 (**43**, Figure 4)	*D. biseptata*	Squalene synthase inhibition	[136,137]
Unknown	Ptr ToxC	*P. tritici-repentis*	Phytotoxic to wheat	[61]

## Data Availability

The data presented in this study are available in this article.

## References

[1] Index Fungorum. http://www.indexfungorum.org.

[2] Zhang G., Berbee M.L. (2001). *Pyrenophora* phylogenetics inferred from ITS and glyceradehyde-3-phosphate dehydrogenase gene sequences. Mycologia.

[3] Kodsueb R., Dhanasekaran V., Aptroot A., Lumyong S., McKenzie E.H., Hyde K.D., Jeewon R. (2006). The family Pleosporaceae: Intergeneric relationships and phylogenetic perspectives based on sequence analyses of partial 28S rDNA. Mycologia.

[4] Ariyawansa H.A., Thambugala K.M., Manamgoda D.S., Jayawardena R., Camporesi E., Boonmee S., Wanasinghe D.N., Phookamsak R., Hongsanan S., Singtripop C. (2015). Towards a natural classification and backbone tree for Pleosporaceae. Fungal Divers..

[5] Ciuffetti L.M., Manning V.A., Pandelova I., Faris J.D., Friesen T.L., Strelkov S.E., Weber G.L., Goodwin S.B., Wolpert T.J., Figueroa M., Dean R.A., Lichens-Park A., Kole C. (2014). *Pyrenophora tritici-repentis*: A plant pathogenic fungus with global impact. Genomics of Plant-Associated Fungi: Monocot Pathogens.

[6] Clare S.J., Wyatt N.A., Brueggeman R.S., Friesen T.L. (2020). Research advances in the *Pyrenophora teres*–barley interaction. Mol. Plant Pathol..

[7] Backes A., Guerriero G., Ait Barka E., Jacquard C. (2021). *Pyrenophora teres*: Taxonomy, morphology, interaction with barley, and mode of control. Front. Plant Sci..

[8] Meyer S.E., Nelson D.L., Clement S., Beckstead J., Kitchen S.G., Pendleton R.L., Monaco T.A., Vernon J.C. (2008). Cheatgrass (*Bromus tectorum*) biocontrol using indigenous fungal pathogens. Proceedings-Shrublands under Fire: Disturbance and Recovery in a Changing World, Cedar City, UT, USA, 6–8 June 2006.

[9] Meyer S.E., Beckstead J., Pearce J., Germino M.J., Chambers J.C., Brown C.S. (2016). Community ecology of fungal pathogens on Bromus tectorum. Exotic Brome-Grasses in Arid and Semiarid Ecosystems of the Western US: Causes, Consequences, and Management Implications.

[10] Akhavan A., Turkington T., Askarian H., Tekauz A., Xi K., Tucker J.R., Kutcher H.R., Strelkov S.E. (2016). Virulence of *Pyrenophora teres* populations in western Canada. Can. J. Plant Pathol..

[11] Tekauz A. (1985). A numerical scale to classify reactions of barley to *Pyrenophora teres*. Can. J. Plant Pathol..

[12] Liu Z., Ellwood S.R., Oliver R.P., Friesen T.L. (2011). *Pyrenophora teres*: Profile of an increasingly damaging barley pathogen. Mol. Plant Pathol..

[13] Moolhuijzen P.M., Muria-Gonzalez M.J., Syme R., Rawlinson C., See P.T., Moffat C.S., Ellwood S.R. (2020). Expansion and conservation of biosynthetic gene clusters in pathogenic *Pyrenophora* spp.. Toxins.

[14] Smedegård-Petersen V. (1977). Isolation of two toxins produced by *Pyrenophora teres* and their significance in disease development of net-spot blotch of barley. Physiol. Plant Pathol..

[15] Bach E., Christensen S., Dalgaard L., Larsen P.O., Olsen C.E., Smedegård-Petersen V. (1979). Structures, properties and relationship to the aspergillomarasmines of toxins produced by *Pyrenophora teres*. Physiol. Plant Pathol..

[16] Friis P., Olsen C.E., Møller B.L. (1991). Toxin production in *Pyrenophora teres*, the ascomycete causing the net-spot blotch disease of barley (*Hordeum vulgare* L.). J. Biol. Chem..

[17] Haenni A.L., Robert M., Vetter W., Roux L., Barbier M., Lederer E. (1965). Structure chimique des aspergillomarasmines A et B. Helv. Chim. Acta.

[18] Arai K., Ashikawa N., Nakakita Y., Matsuura A., Ashizawa N., Munekata M. (1993). Aspergillomarasmine A and B, potent microbial inhibitors of endothelin-converting enzyme. Biosci. Biotechnol. Biochem.

[19] Liao D., Yang S., Wang J., Zhang J., Hong B., Wu F., Lei X. (2016). Total synthesis and structural reassignment of aspergillomarasmine A. Angew. Chem..

[20] Albu S.A., Koteva K., King A.M., Al-Karmi S., Wright G.D., Capretta A. (2016). Total synthesis of aspergillomarasmine A and related compounds: A sulfamidate approach enables exploration of structure–activity relationships. Angew. Chem..

[21] Koteva K., King A.M., Capretta A., Wright G.D. (2016). Total synthesis and activity of the metallo-β-lactamase inhibitor aspergillomarasmine A. Angew. Chem. Int. Ed..

[22] Zhang J., Wang S., Bai Y., Guo Q., Zhou J., Lei X. (2017). Total syntheses of natural metallophores staphylopine and aspergillomarasmine A. J. Org. Chem..

[23] Fu H., Zhang J., Saifuddin M., Cruiming G., Tepper P.G., Poelarends G.J. (2018). Chemoenzymatic asymmetric synthesis of the metallo-β-lactamase inhibitor aspergillomarasmine A and related aminocarboxylic acids. Nat. Catal..

[24] King A.M., Reid-Yu S.A., Wang W., King D.T., De Pascale G., Strynadka N.C., Walsh T.R., Coombes B.K., Wright G.D. (2014). Aspergillomarasmine A overcomes metallo-β-lactamase antibiotic resistance. Nature.

[25] Sychantha D., Rotondo C.M., Tehrani K.H., Martin N.I., Wright G.D. (2021). Aspergillomarasmine A inhibits metallo-β-lactamases by selectively sequestering Zn^2+^. J. Biol. Chem..

[26] Weiergang I., Jørgensen H.L., Møller I.M., Friis P., Smedegaard-Petersen V. (2002). Correlation between sensitivity of barley to *Pyrenophora teres* toxins and susceptibility to the fungus. Physiol. Mol. Plant Pathol..

[27] Sarpeleh A., Tate M.E., Wallwork H., Catcheside D., Able A.J. (2008). Characterisation of low molecular weight phytotoxins isolated from *Pyrenophora teres*. Physiol. Mol. Plant Pathol..

[28] Ballio A., Bottalico A., Buonocore V., Carilli A., Di Vittorio V., Graniti A. (1969). Production and isolation of aspergillomarasmin B (lycomarasmic acid) from cultures of *Colletotrichum gloeosporioides* Penz. (*Gloeosporium olivarum* Aim.). Phytopathol. Mediterr..

[29] Nukina M., Ikeda M., Sassa T. (1980). Two new pyrenolides, fungal morphogenic substances produced by *Pyrenophora teres* (Diedicke) Drechsler. Agric. Biol. Chem..

[30] Nukina M., Sassa T., Ikeda M. (1980). A new fungal morphogenic substance, pyrenolide A from *Pyrenophora teres*. Tetrahedron Lett..

[31] Venkatasubbaiah P., Chilton W.S. (1992). Phytotoxins of *Ascochyta hyalospora*, causal agent of lambsquarters leaf spot. J. Nat. Prod..

[32] Greve H., Schupp P.J., Eguereva E., Kehraus S., König G.M. (2008). Ten-membered lactones from the marine-derived fungus *Curvularia* sp.. J. Nat. Prod..

[33] Asaoka M., Naito S., Takei H. (1985). Total synthesis of (±)-pyrenolide B. Tetrahedron Lett..

[34] Suzuki S., Tanaka A., Yamashita K. (1987). Synthesis and biological activity of (+)-pyrenolide B. Agric. Biol. Chem..

[35] Moricz A., Gassmann E., Bienz S., Hesse M. (1995). Synthesis of (±)-pyrenolide B. Helv. Chim. Acta.

[36] Wasserman H.H., Prowse K.S. (1992). The singlet oxygen conversion of oxazoles to triamides. Application in the synthesis of (±)-pyrenolide C. Assignment of stereochemistry. Tetrahedron.

[37] Nukina M., Hirota H. (1992). Pyrenolide D, a new cytotoxic fungal metabolite from *Pyrenophora teres*. Biosci. Biotechnol. Biochem..

[38] Engstrom K.M., Mendoza M.R., Navarro-Villalobos M., Gin D.Y. (2001). Total synthesis of (+)-pyrenolide D. Angew. Chem. Int. Ed..

[39] Thirupathi B., Reddy P.P., Mohapatra D.K. (2011). A carbohydrate-based total syntheses of (+)-pyrenolide D and (−)-4-*epi*-pyrenolide D. J. Org. Chem..

[40] Zhang C., Liu J., Du Y. (2013). A concise total synthesis of (+)-pyrenolide D. Tetrahedron Lett..

[41] Markovič M., Lopatka P., Koóš P., Gracza T. (2014). Asymmetric formal synthesis of (+)-pyrenolide D. Synthesis.

[42] Ogawa Y., Kato M., Sasaki I., Sugimura H. (2018). Total synthesis of (+)-pyrenolide D. J. Org. Chem..

[43] Coval S.J., Hradil C.M., Lu H.S., Clardy J., Satouri S., Strobel G.A. (1990). Pyrenoline-A and-B, two new phytotoxins from *Pyrenophora teres*. Tetrahedron Lett..

[44] Benali D., Lyamani A., Zaid A., Samih M., Haloui N. (1995). Cinétique de production de toxines de type pyrenoline A et pyrenoline B par des isolats marocains de *Pyrenophora teres*. Phytopathol. Mediterr..

[45] Engström K., Brishammar S., Svensson C., Bengtsson M., Andersson R. (1993). Anthraquinones from some *Drechslera* species and *Bipolaris sorokiniana*. Mycol. Res..

[46] Wakuliński W., Kachlicki P., Sobiczewski P., Schollenberger M., Zamorski C., Łotocka B., Sarova J. (2003). Catenarin production by isolates of *Pyrenophora tritici-repentis* (Died.) Drechsler and its antimicrobial activity. J. Phytopathol..

[47] Sadorn K., Saepua S., Boonyuen N., Komwijit S., Rachtawee P., Pittayakhajonwut P. (2019). Phenolic glucosides and chromane analogs from the insect fungus *Conoideocrella krungchingensis* BCC53666. Tetrahedron.

[48] Anslow W.K., Raistrick H. (1941). Synthesis of catenarin (1:4:5:7-tetrahydroxy-2-methylanthraquinone), a metabolic product of species of *Helminthosporium*. Biochem..

[49] Chandrasenan K., Neelakantan S., Seshadri T.R. (1960). A new synthesis of catenarin and erythroglaucin. Proc. Indian Natl. Sci. Acad..

[50] Bouras N., Strelkov S.E. (2008). The anthraquinone catenarin is phytotoxic and produced in leaves and kernels of wheat infected by *Pyrenophora tritici-repentis*. Physiol. Mol. Plant Pathol..

[51] Martorell M., Castro N., Victoriano M., Capó X., Tejada S., Vitalini S., Pezzani R., Sureda A. (2021). An update of anthraquinone derivatives emodin, diacerein, and catenarin in diabetes. Evid. Based Complementary Altern. Med..

[52] Mehrabi R., Bahkali A.H., Abd-Elsalam K.A., Moslem M., M’Barek S.B., Gohari A.M., Jashni M.K., Stergiopoulos I., Kema G.H.J., de Wit P.J.G.M. (2011). Horizontal gene and chromosome transfer in plant pathogenic fungi affecting host range. FEMS Microbiol. Rev..

[53] Friesen T.L., Stukenbrock E.H., Liu Z.H., Meinhardt S., Ling H., Faris J.D., Rasmussen J.B., Solomon P.S., McDonald B.A., Oliver R.P. (2006). Emergence of a new disease as a result of interspecific virulence gene transfer. Nat. Genet..

[54] Antoni E.A., Rybak K., Tucker M.P., Hane J.K., Solomon P.S., Drenth A., Shankar M., Oliver R.P. (2010). Ubiquity of ToxA and absence of ToxB in Australian populations of *Pyrenophora tritici-repentis*. Australas. Plant Pathol..

[55] Leisova-Syobodova L., Hanzalova A., Kucera L. (2010). Expansion and variability of the Ptr Tox A gene in populations of *Pyrenophora tritici-repentis* and *Pyrenophora teres*. J. Plant Pathol..

[56] Rawlinson C., See P.T., Moolhuijzen P., Li H., Moffat C.S., Chooi Y.H., Oliver R.P. (2019). The identification and deletion of the polyketide synthase-nonribosomal peptide synthase gene responsible for the production of the phytotoxic triticone A/B in the wheat fungal pathogen *Pyrenophora tritici**-repentis*. Environ. Microbiol..

[57] Ballance G.M., Lamari L., Bernier C.C. (1989). Purification and characterization of a host-selective necrosis toxin from *Pyrenophora tritici-repentis*. Physiol. Mol. Plant Pathol..

[58] Strelkov S.E., Lamari L., Ballance G.M. (1999). Characterization of a host-specific protein toxin (Ptr ToxB) from *Pyrenophora tritici-repentis*. Mol. Plant-Microbe Interact..

[59] Pandelova I., Figueroa M., Wilhelm L.J., Manning V.A., Mankaney A.N., Mockler T.C., Ciuffetti L.M. (2012). Host-selective toxins of *Pyrenophora tritici-repentis* induce common responses associated with host susceptibility. PLoS ONE.

[60] Andrie R.M., Schoch C.L., Hedges R., Spatafora J.W., Ciuffetti L.M. (2008). Homologs of ToxB, a host-selective toxin gene from *Pyrenophora tritici-repentis*, are present in the genome of sister-species *Pyrenophora bromi* and other members of the Ascomycota. Fungal Genet. Biol..

[61] Effertz R.J., Meinhardt S.W., Anderson J.A., Jordahl J.G., Francl L.J. (2002). Identification of a chlorosis-inducing toxin from *Pyrenophora tritici-repentis* and the chromosomal location of an insensitivity locus in wheat. Phytopathology.

[62] Betts M.F., Manning V.A., Cardwell K.B., Pandelova I., Ciuffetti L.M. (2011). The importance of the N-terminus for activity of Ptr ToxB, a chlorosis-inducing host-selective toxin produced by *Pyrenophora tritici-repentis*. Physiol. Mol. Plant Pathol..

[63] Shi G., Kariyawasam G., Liu S., Leng Y., Zhong S., Ali S., Moolhuijzen P., Moffat C.S., Rasmussen J.B., Friesen T.L. (2022). A conserved hypothetical gene is required but not sufficient for Ptr ToxC production in *Pyrenophora tritici-repentis*. Mol. Plant-Microbe Interact..

[64] Hallock Y.F., Lu H.S., Clardy J., Strobel G.A., Sugawara F., Samsoedin R., Yoshida S. (1993). Triticones, spirocyclic lactams from the fungal plant pathogen *Drechslera tritici-repentis*. J. Nat. Prod..

[65] Sugawara F., Takahashi N., Strobel G.A., Strobel S.A., Lu H.S., Clardy J. (1988). Triticones A and B, novel phytotoxins from the plant pathogenic fungus *Drechslera tritici-repentis*. J. Am. Chem. Soc..

[66] Kenfield D., Strobel S., Sugawara F., Berglund D., Strobel G. (1988). Triticone A: A novel bioactive lactam with potential as a molecular probe. Biochem. Biophys. Res. Commun..

[67] Shinohara C., Chikanishi T., Nakashima S., Hashimoto A., Hamanaka A., Endo A., Hasumi K. (2000). Enhancement of fibrinolytic activity of vascular endothelial cells by chaetoglobosin A, crinipellin B, geodin and triticone B. J. Antibiot..

[68] Hilario F., Polinário G., de Amorim M.R., de Sousa Batista V., do Nascimento N.M., Araújo A.R., Baubab T.M., Dos Santos L.C. (2020). Spirocyclic lactams and curvulinic acid derivatives from the endophytic fungus *Curvularia lunata* and their antibacterial and antifungal activities. Fitoterapia.

[69] Bouras N., Strelkov S.E. (2010). Influence of carbon source on growth and mycotoxin production by isolates of *Pyrenophora tritici-repentis* from wheat. Can. J. Microbiol..

[70] Meyer S.E., Quinney D., Nelson D.L., Weaver J. (2007). Impact of the pathogen *Pyrenophora semeniperda* on *Bromus tectorum* seedbank dynamics in North American cold deserts. Weed Res..

[71] Paul A.R. (1969). The production of *Pyrenophora semeniperda* in culture. Trans. Br. Mycol. Soc..

[72] Medd R.W., Murray G.M., Pickering D.I. (2003). Review of the epidemiology and economic importance of *Pyrenophora semeniperda*. Australas. Plant Pathol..

[73] Medd R.W., Campbell M.A. (2005). Grass seed infection following inundation with *Pyrenophora semeniperda*. Biocontrol. Sci. Technol..

[74] Beckstead J., Meyer S.E., Molder C.J., Smith C. (2007). A race for survival: Can *Bromus tectorum* seeds escape *Pyrenophora semeniperda*-caused mortality by germinating quickly?. Ann. Bot..

[75] Allen P.S., Finch-Boekweg H., Meyer S.E. (2018). A proposed mechanism for high pathogen-caused mortality in the seed bank of an invasive annual grass. Fungal Ecol..

[76] Meyer S.E., Stewart T.E., Clement S. (2010). The quick and the deadly: Growth vs virulence in a seed bank pathogen. New Phytol..

[77] Masi M., Evidente A., Meyer S., Nicholson J., Muñoz A. (2014). Effect of strain and cultural conditions on the production of cytochalasin B by the potential mycoherbicide *Pyrenophora semeniperda* (Pleosporaceae, Pleosporales). Biocontrol. Sci. Technol..

[78] Meyer S.E., Masi M., Clement S., Davis T.L., Beckstead J. (2015). Mycelial growth rate and toxin production in the seed pathogen *Pyrenophora semeniperda*: Resource trade-offs and temporally varying selection. Plant Pathol..

[79] Boose D., Harrison S., Clement S., Meyer S. (2011). Population genetic structure of the seed pathogen *Pyrenophora semeniperda* on *Bromus tectorum* in western North America. Mycologia.

[80] Coleman C.E., Meyer S.E., Ricks N. (2019). Mating system complexity and cryptic speciation in the seed bank pathogen *Pyrenophora semeniperda*. Plant Pathol..

[81] Beckstead J., Meyer S.E., Reinhart K.O., Bergen K.M., Holden S.R., Boekweg H.F. (2014). Factors affecting host range in a generalist seed pathogen of semi-arid shrublands. Plant Ecol..

[82] Beckstead J., Meyer S.E., Ishizuka T.S., McEvoy K.M., Coleman C.E. (2016). Lack of host specialization on winter annual grasses in the fungal seed bank pathogen *Pyrenophora semeniperda*. PLoS ONE.

[83] Soliai M.M., Meyer S.E., Udall J.A., Elzinga D.E., Hermansen R.A., Bodily P.M., Hart A.A., Coleman C.E. (2014). De novo genome assembly of the fungal plant pathogen *Pyrenophora semeniperda*. PLoS ONE.

[84] Evidente A., Andolfi A., Vurro M., Zonno M.C., Motta A. (2002). Cytochalasins Z1, Z2 and Z3, three 24-oxa[14]cytochalasans produced by *Pyrenophora semeniperda*. Phytochemistry.

[85] Aldridge D.C., Armstrong J.J., Speake R.N., Turner W.B. (1967). The cytochalasins, a new class of biologically active mould metabolites. Chem. Comm..

[86] Scherlach K., Boettger D., Remme N., Hertweck C. (2010). The chemistry and biology of cytochalasans. Nat. Prod. Rep..

[87] Campbell M.A., Medd R.W., Brown J.B. (2003). Optimizing conditions for growth and sporulation of *Pyrenophora semeniperda*. Plant Pathol..

[88] Masi M., Meyer S., Clement S., Andolfi A., Cimmino A., Evidente A. (2014). Spirostaphylotrichin W, a spirocyclic γ-lactam isolated from liquid culture of *Pyrenophora semeniperda*, a potential mycoherbicide for cheatgrass (*Bromus tectorum*) biocontrol. Tetrahedron.

[89] Masi M., Meyer S., Cimmino A., Andolfi A., Evidente A. (2014). Pyrenophoric acid, a phytotoxic sesquiterpenoid penta-2,4-dienoic acid produced by a potential mycoherbicide, *Pyrenophora semeniperda*. J. Nat. Prod..

[90] Cimmino A., Masi M., Evidente M., Superchi S., Evidente A. (2017). Application of Mosher’s method for absolute configuration assignment to bioactive plants and fungi metabolites. J. Pharm. Biomed. Anal..

[91] Masi M., Meyer S., Cimmino A., Clement S., Black B., Evidente A. (2014). Pyrenophoric acids B and C, two new phytotoxic sesquiterpenoids produced by *Pyrenophora semeniperda*. J. Agric. Food Chem..

[92] Lozano-Juste J., Masi M., Cimmino A., Clement S., Fernández M.A., Antoni R., Meyer S., Rodriguez P.L., Evidente A. (2019). The fungal sesquiterpenoid pyrenophoric acid B uses the plant ABA biosynthetic pathway to inhibit seed germination. J. Exp. Bot..

[93] da Rosa C.R., Martinelli J.A., Federizzi L.C., Bocchese C.A. (2003). Quantification of conidia produced by *Pyrenophora chaetomioides* on dead leaves of *Avena sativa* under field condition. Fitopatol. Bras..

[94] Chen H., Xue L., White J.F., Kamran M., Li C. (2022). Identification and characterization of *Pyrenophora* species causing leaf spot on oat (*Avena sativa*) in western China. Plant Pathol..

[95] Lam A. (1984). *Drechslera siccans* from ryegrass fields in England and Wales. Trans. Br. Mycol. Soc..

[96] Wiewióra B., Żurek G., Żurek M. (2015). Endophyte-mediated disease resistance in wild populations of perennial ryegrass (*Lolium perenne*). Fungal Ecol..

[97] Osterhage C., König G.M., Höller U., Wright A.D. (2002). Rare sesquiterpenes from the algicolous fungus *Drechslera dematioidea*. J. Nat. Prod..

[98] Shoemaker R.A. (1998). *Marielliottia*, a new genus of cereal and grass parasites segregated from *Drechslera*. Can. J. Bot..

[99] Jones E.B.G., Sakayaroj J., Suetrong S., Somrithipol S., Pang K.L. (2009). Classification of marine Ascomycota, anamorphic taxa and Basidiomycota. Fungal Divers..

[100] Nozoe S., Hirai K., Tsuda K., Ishibashi K., Shirasaka M., Grove J.F. (1965). The structure of pyrenophorin. Tetrahedron Lett..

[101] Sugawara F., Strobel G.A. (1986). (−)-Dihydropyrenophorin, a novel and selective phytotoxin produced by *Drechslera avenae*. Plant Sci..

[102] Lerario P., Graniti A. (1985). Attività fitotossica della pirenoforina e sua produzione nelle colture di *Pyrenophora avenae* Ito *et* Kurib. Phytopathol. Mediterr..

[103] McMullin D.R., Green B.D., Miller J.D. (2015). Antifungal sesquiterpenoids and macrolides from an endophytic *Lophodermium* species of *Pinus strobus*. Phytochemistry Lett..

[104] Yu H., Sperlich J., Höfert S.P., Janiak C., Teusch N., Stuhldreier F., Wesselborg S., Wang C., Kassack M.U., Dai H. (2019). Azaphilone pigments and macrodiolides from the coprophilous fungus *Coniella fragariae*. Fitoterapia.

[105] Ramakrishna K., Sreenivasulu R., Vidavalur S., Jagan Mohan Reddy B. (2016). Stereoselective total synthesis of (-)-pyrenophorin. Lett. Org. Chem..

[106] Zhang W., Krohn K., Egold H., Draeger S., Schulz B. (2008). Diversity of antimicrobial pyrenophorol derivatives from an endophytic fungus, *Phoma* sp.. Eur. J. Org. Chem..

[107] Kastanias M.A., Chrysayi-Tokousbalides M. (2000). Herbicidal potential of pyrenophorol isolated from a *Drechslera avenae* pathotype. Pest Manag. Sci..

[108] Yadav J.S., Reddy U.S., Reddy B.S. (2009). Stereoselective total synthesis of (−)-pyrenophorol. Tetrahedron Lett..

[109] Raistrick H., Robinson R., Todd A.R. (1934). Studies in the biochemistry of micro-organisms: (a) On the production of hydroxyanthraquinones by species of *Helminthosporium*. (b) Isolation of tritisporin, a new metabolic product of *Helminthosporium tritici-vulgaris* Nisikado. (c) The molecular constitution of catenarin. Biochem. J..

[110] Shujun J., Sheng Q., Yunzhi Z. (2006). Isolation, purification, identification, and bioassay of helminthosporin with herbicidal activity from *Curvularia eragrostidis*. Acta Phytophylacica Sin..

[111] Fozia A.A. (2014). Phytochemical Investigation of *Aloe turkanensis* for Anticancer Activity. Doctoral Dissertation.

[112] Augustin N., Nuthakki V.K., Abdullaha M., Hassan Q.P., Gandhi S.G., Bharate S.B. (2020). Discovery of helminthosporin, an anthraquinone isolated from *Rumex abyssinicus* Jacq as a dual cholinesterase inhibitor. ACS Omega.

[113] Chrysayi-Tokousbalides M., Kastanias M.A. (2003). Cynodontin: A fungal metabolite with antifungal properties. J. Agric. Food Chem..

[114] Đorović J., Antonijević M., Marković Z. (2020). Antioxidative and inhibition potency of cynodontin. J. Serb. Soc. Comput. Mech..

[115] van Eijk G.W. (1974). Chrysophanol and emodin from *Drechslera catenaria*. Phytochemistry.

[116] Dussart F., Jakubczyk D. (2022). Biosynthesis of rubellins in *Ramularia collo-cygni*—Genetic basis and pathway proposition. Int. J. Mol. Sci..

[117] Choi G.J., Lee S.W., Jang K.S., Kim J.S., Cho K.Y., Kim J.C. (2004). Effects of chrysophanol, parietin, and nepodin of *Rumex crispus* on barley and cucumber powdery mildews. Crop Prot..

[118] Yusuf M.A., Singh B.N., Sudheer S., Kharwar R.N., Siddiqui S., Abdel-Azeem A.M., Fraceto L.F., Dashora K., Gupta V.K. (2019). Chrysophanol: A natural anthraquinone with multifaceted biotherapeutic potential. Biomolecules.

[119] Su S., Wu J., Gao Y., Luo Y., Yang D., Wang P. (2020). The pharmacological properties of chrysophanol, the recent advances. Biomed. Pharmacother..

[120] Lin C.H., Tseng H.F., Hsieh P.C., Chiu V., Lin T.Y., Lan C.C., Tzeng I.S., Chao H.N., Hsu C.C., Kuo C.Y. (2021). Nephroprotective role of chrysophanol in hypoxia/reoxygenation-induced renal cell damage via apoptosis, ER stress, and ferroptosis. Biomedicines.

[121] Jadaun K.S., Mehan S., Sharma A., Siddiqui E.M., Kumar S., Alsuhaymi N. (2022). Neuroprotective effect of chrysophanol as a PI3K/AKT/mTOR signaling inhibitor in an experimental model of autologous blood-induced intracerebral hemorrhage. Curr. Med. Sci..

[122] Cui W.H., Zhang H.H., Qu Z.M., Wang Z., Zhang D.J., Wang S. (2022). Effects of chrysophanol on hippocampal damage and mitochondrial autophagy in mice with cerebral ischemia reperfusion. Int. J. Neurosci..

[123] Dong X., Fu J., Yin X., Cao S., Li X., Lin L., Huyiligeqi, Ni J. (2016). Emodin: A review of its pharmacology, toxicity and pharmacokinetics. Phytother. Res..

[124] Anke H., Kolthoum I., Laatsch H. (1980). Metabolic products of microorganisms. 192. The anthraquinones of the *Aspergillus glaucus* group. II. Biological activity. Arch. Microbiol..

[125] Hasan H.A.H. (1998). Studies on toxigenic fungi in roasted foodstuff (salted seed) and halotolerant activity of emodin-producing *Aspergillus wentii*. Folia Microbiol..

[126] Macías M., Ulloa M., Gamboa A., Mata R. (2000). Phytotoxic compounds from the new coprophilous fungus *Guanomyces polythrix*. J. Nat. Prod..

[127] Hallock Y.F., Clardy J., Kenfield D.S., Strobel G. (1988). De-*O*-methyldiaporthin, a phytotoxin from *Drechslera siccans*. Phytochemistry.

[128] Evidente A., Andolfi A., Vurro M., Fracchiolla M., Zonno M.C., Motta A. (2005). Drazepinone, a trisubstituted tetrahydronaphthofuroazepinone with herbicidal activity produced by *Drechslera siccans*. Phytochemistry.

[129] Cao F., Pan L., Gao W., Liu Y., Zheng C., Zhang Y. (2021). Structure revision and protein tyrosine phosphatase inhibitory activity of drazepinone. Mar. Drugs.

[130] Lim C.H., Miyagawa H., Ueno T., Takenaka H., Sung N.D. (1996). Siccanol: Sesterterpene isolated from pathogenic fungus *Drechslera siccans*. Appl. Biol. Chem..

[131] Chan J., Jamison T.F. (2004). Enantioselective synthesis of (−)-terpestacin and structural revision of siccanol using catalytic stereoselective fragment couplings and macrocyclizations. J. Am. Chem. Soc..

[132] Masi M., Zonno M.C., Boari A., Vurro M., Evidente A. (2022). Terpestacin, a toxin produced by *Phoma exigua* var. *heteromorpha*, the causal agent of a severe foliar disease of oleander (*Nerium oleander* L.). Nat. Prod. Res..

[133] Ishibashi K. (1962). Studies on antibiotics from *Helminthosporium* sp. fungi. VII Siccanin, a new antifungal antibiotic produced by *Helminthosporium siccans*. J. Antibiot..

[134] Mogi T., Kawakami T., Arai H., Igarashi Y., Matsushita K., Mori M., Shiomi K., Ōmura S., Harada S., Kita K. (2009). Siccanin rediscovered as a species-selective succinate dehydrogenase inhibitor. J. Biochem..

[135] Trost B.M., Shen H.C., Surivet J.P. (2003). An enantioselective biomimetic total synthesis of (−)-siccanin. Angew. Chem..

[136] Bills G.F., Peláez F., Polishook J.D., Diez-Matas M.T., Harris G.H., Clapp W.H., Dufresne C., Byrne K.M., Nallin-Omstead M., Jenkins R.G. (1994). Distribution of zaragozic acids (squalestatins) among filamentous ascomycetes. Mycol. Res..

[137] Huang L., Lingham R.B., Harris G.H., Singh S.B., Dufresne C., Nallin-Omstead M., Bills G.F., Mojena M., Sanchez M., Karkas J.D. (1995). New fungal metabolites as potential antihypercholesterolemics and anticancer agents. Can. J. Bot..

[138] Nicolaou K.C., Yue E.W., La Greca S., Nadin A., Yang Z., Leresche J.E., Tsuri T., Naniwa Y., de Riccardis F. (1995). Synthesis of zaragozic acid A/squalestatin S1. Eur. J. Chem..

[139] Sarpeleh A., Wallwork H., Catcheside D.E., Tate M.E., Able A.J. (2007). Proteinaceous metabolites from *Pyrenophora teres* contribute to symptom development of barley net blotch. Phytopathology.

[140] Van Goietsenoven G., Mathieu V., Andolfi A., Cimmino A., Lefranc F., Kiss R., Evidente A. (2011). In vitro growth inhibitory effects of cytochalasins and derivatives in cancer cells. Planta Med..

[141] Berestetskiy A., Dmitriev A., Mitina G., Lisker I., Andolfi A., Evidente A. (2008). Nonenolides and cytochalasins with phytotoxic activity against *Cirsium arvense* and *Sonchus arvensis*: A structure–activity relationships study. Phytochemistry.

[142] Cimmino A., Andolfi A., Berestetskiy A., Evidente A. (2008). Production of phytotoxins by *Phoma exigua* var. exigua, a potential mycoherbicide against perennial thistles. J. Agric. Food Chem..

[143] Sumarah M.W., Kesting J.R., Sørensen D., Miller J.D. (2011). Antifungal metabolites from fungal endophytes of *Pinus strobus*. Phytochemistry.

[144] Chooi Y.H., Solomon P.S. (2014). A chemical ecogenomics approach to understand the roles of secondary metabolites in fungal cereal pathogens. Front. Microbiol..

